# Changes in Household Dietary Diversity in Herder Communities over the Past 20 Years: Evidence from Xilin Gol Grassland of China

**DOI:** 10.3390/foods12112271

**Published:** 2023-06-05

**Authors:** Wanni Yang, Lin Zhen, Yunjie Wei

**Affiliations:** 1 China Center for Agricultural Policy, School of Advanced Agricultural Sciences, Peking University, No. 5 Yiheyuan Road, Haidian District, Beijing 100871, China; wanniyang@pku.edu.cn; 2 Institute of Geographic Sciences and Natural Resources Research, Chinese Academy of Sciences, No. 11A Datun Road, Chaoyang District, Beijing 100101, China; 3 College of Resources and Environment, University of Chinese Academy of Sciences, A19 Yuquan Road, Shijingshan District, Beijing 100049, China; 4 School of Economics, Beijing Technology and Business University, No. 33 Fucheng Road, Haidian District, Beijing 100048, China; weiyunjie@btbu.edu.cn

**Keywords:** household dietary diversity, animal-based and plant-based food, herder community, grassland areas, northern China

## Abstract

Food security is critical for socioeconomic development. In grassland areas, inappropriate food consumption patterns can cause irreversible damage to vulnerable local ecosystems. This study aims to examine the household dietary diversity status and development trend over the past 20 years in Chinese herder communities. We draw on a cross-sectional dataset of 230 households involving 652 family members from the Xilin Gol Grassland areas in North China. Household dietary diversity was assessed using the household dietary diversity score (HDDS), which was calculated based on 12 food groups. Results show that HDDS increased from 3.74 in 1999 to 5.92 in 2019, with an annual average growth rate of 2.45% during the past 20 years. The increase in plant-based food scores made a major contribution to the HDDS improvement. The variations in household dietary diversity status between pastoral areas and agro-pastoral areas showed differences among different types of grassland in arid and semiarid transitional zones. It is worth paying more attention to monitoring the main impact factors that affect HDDS and how these changes might impact the local ecosystem, which will benefit regional sustainable development.

## 1. Introduction

As one of the Sustainable Development Goals (SDGs), food security is critical for socio-economic development [1] and is an important strategic foundation for regional development [2]. With socioeconomic development and population growth, societies’ food consumption patterns have also changed. Factors such as the development of transportation [3,4], online shopping [5,6], the increase in income [7,8], awareness of healthy food [9,10], and so on, have had effects on populations’ daily food consumption status. These changes are also taking place in herder communities. During the past 20 years, herders in northern China have turned their lifestyle from a nomadic one to a settled one [11]. Therefore, an evolution of lifestyle and production activity patterns has been occurred in those areas. For example, their food supply conditions have become more diverse [12], their accessibility to markets has been improved [13], and planting in private gardens could be a possible family choice [14]. Under the changes in the food supply environment, the question “What does the household food consumption style look like?” arises in our minds.

Previous studies showed that food consumption in the grassland region is complex [15,16]. Inappropriate anthropogenic activities (i.e., overgrazing, excessive reclamation, etc.) negatively affect local grassland ecosystems [17], and many resultant effects are either be irreversible or necessitate considerable restoration costs [18,19]. Household food consumption activity is one of those actions that increases pressure on local environmental carrying capacity [20], which may lead to irreversible impacts on the grassland ecosystem [21,22]. Increasing food demand would encourage unsustainable resource use behaviors, such as over-grazing, over-farming, and the indiscriminate use of fertilizers [23], so that limited local ecological resources could meet the aggregated food demand [21]. Understanding household dietary diversity status and its development trends will help us to better understand the local ecosystem [24] and build a safer social net, which might have a stronger ability and better resilience to resist socio-economic crises [25]. For example, the policymakers should target and purposefully to monitor the factors that lead to high-resource-required dietary patterns and provide prompt and necessary interventions; additionally, policymakers could be better prepared in terms of food supply when the regional household dietary status displays negative signs due to natural disasters.

This study used Xilin Gol Grassland as a case study to explore the household dietary status in herder communities. Compared to cultivated farming communities, herder communities usually rely on grassland ecosystems, which are located in semi-arid areas. The irrational use of natural resources would lead to irreversible damage to the grassland system. Due to its sensitive, vulnerable environment, as well as appreciable human activity, this region faces significant challenges in regard to sustainability [26,27]. Food consumption behavior is one of the activities that we need to pay attention to in this region [22]. Local and central governments have invested over CNY 200 million into regional ecological projects. However, the limited cultivated land distributed within the southern border of the Xilin Gol Grassland produces a limited supply of food, which can only meet all regional needs with difficulty [28]. Previous studies in agro-pastoral areas show that traditional herder communities had less diverse dietary structures and that the animal-based food consumption and demand kept rising [29,30]. However, this demand led to the irrational utilization of rangeland and the accelerated utilization of direct and indirect water resources, which placed huge pressure on local ecosystem sustainability [31,32,33]. 

Although the previous studies have focused on residents’ food consumption changes in semi-arid regions, the food consumption quantity was the spotlight. Household dietary diversity status is one of the most important aspects for assessing household food security status. Moreover, due to the accessibility of the dataset, statistical data were some of the main data sources used to analyze regional household food consumption status. There are a lack of field survey data to depict household dietary diversity status in herder communities. In this study, we tried to use first-hand data to explore household dietary diversity status and its characteristics in the herder community in Xilin Gol Grassland and also tried to uncover the trend of household diet changes during the past 20 years. The results provided the scientific basis for regional development and implications for policymakers to aid in creating regional sustainable development strategies. 

## 2. Materials and Methods

### 2.1. Study Area

The Xilin Gol Grassland is one of the largest grassland areas in China. The agro-pastoral transitional zone in northern China transects through the southern section of the Xilin Gol Grassland. The Xilin Gol League comprises most of the Xilin Gol Grassland, which is within the central Inner Mongolia Autonomous Region, China. It belongs to the Mongolian Plateau, at an altitude of 800–1800 m. Annual precipitation is 288 mm; annual evaporation is between 1700 and 2600 mm; and the annual average temperature is 3.60 °C. It comprises two county-level cities, one county, nine banners (i.e., a county-level administrative entities in northern China), and two districts. At the end of 2019, the total registered population was 1.04 million, of which the rural population accounted for 54.26%. This region comprises multinational ethnic groups, such as the Han (63.69%), Mongolian (31.75%), and Hui, to name the most prevalent. The total land-use area of the Xilin Gol League is 0.2 million km^2^, including 180,000 km^2^ of grassland and 5859.50 km^2^ of forestland, accounting for 89.90% and 7.10% of the total land-use area, respectively. Grassland is the primary land-use type in the Xilin Gol League. Cattle and sheep are the main livestock raised in this region. In 2019, the annual per capita disposable income of residents of the Xilin Gol League was CNY 32,460, of which the per capita annual disposable income of urban residents was CNY 40,778, while the per capita annual disposable income of rural residents was CNY 17,391.

### 2.2. Selection of Study Sites

**Figure 1:**
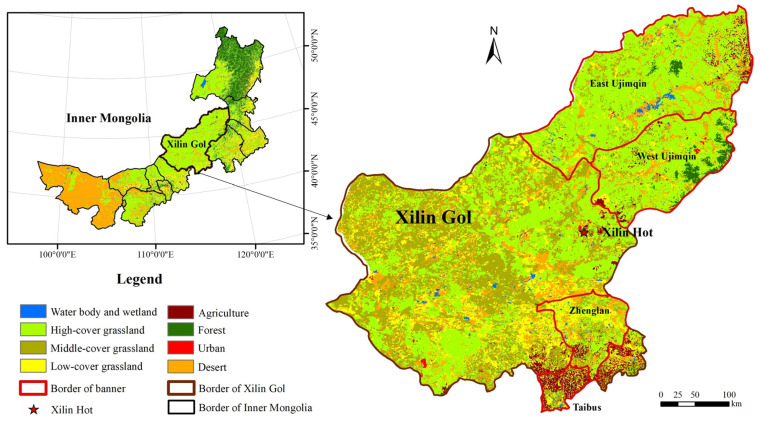
Location of study areas. Data source: The data center for Resource and Environment Data Platform of CAS (http://www.resdc.cn, accessed on 25 April 2023).

The Xilin Gol Grassland is expansive (i.e., 2.03 × 10^5^ km^2^). The land-cover types of the Xilin Gol Grassland can be subdivided as follows: typical grassland, desert grassland, cultivated land, and sporadic grassland. The carrying capacity of these grassland land-use types gradually decreases directionally, exhibiting a gradient change from north to south, which constitutes the Xilin Gol Grassland transects. In this study, we selected four banners located in Xilin Gol Grassland for our investigation: the East Ujimqin Banner and West Ujimqin Banner, the typical pastoral grassland areas; the Zhenglan Banner, a pastoral area of the Hunshandake Sandy Land; and the Taibus Banner, an agro-pastoral transitional zone (Figure 1). The East Ujimqin Banner and West Ujimqin Banner are in the northern region of the Xilin Gol League, while the Zhenglan Banner and the Taibus Banner are in the southern region. 

All banners are located in arid and semiarid transitional zones, namely, where annual precipitation is approximately 400 mm and annual evaporation is extremely high (>1800 mm). Ecosystems within the study areas are considered extremely vulnerable. In these areas, pastoral activities and farming activities coexist. Furthermore, herder communities are facing lifestyle changes due to the changes from nomadic life to settled life in the past 20 years. Hence, local households are facing dietary and lifestyle revolutions.

### 2.3. Data Collection

Data used for this study were gathered from household surveys conducted within the study area from 20 July to 5 August 2019. Pen-and-paper personal interviews (PAPI) were conducted by 5 trained enumerators in a standardized way. Before the formal field survey, all enumerators were trained intensively for one day to ensure a standardized understanding of the survey, followed by a practice run in the field. The interviews were undertaken in Mandarin and Mongolian, depending on the language abilities of the interviewees. Approximately 50% of interviewees required a translator to guarantee that the interviews were conducted successfully, including those who could only understand but could not express themselves fluently in Mandarin. Locals were used as translators, such as officials who worked at the local grassland observation station, head of the village, and young college students.

**Figure 2:**
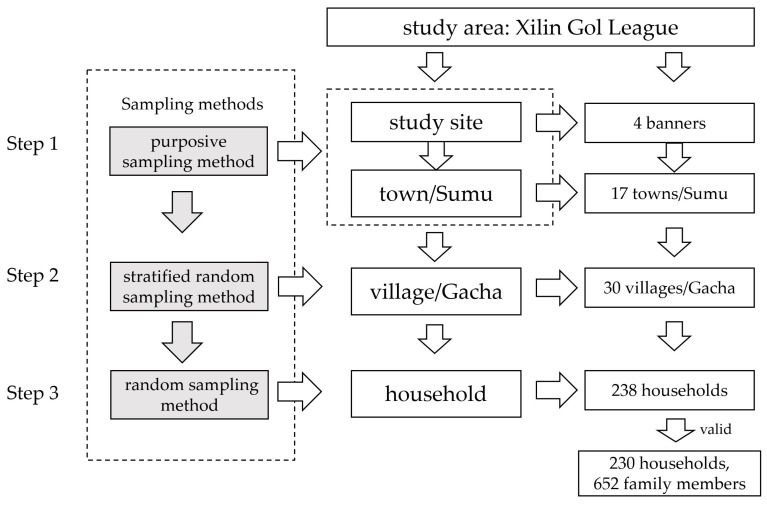
Determination process of the study sample.

For the sampling, we used the purposive sampling method, stratified random sampling method, and random sampling method (Figure 2). In the first step, we used the purposive sampling method to determine the scope of the survey, which refers to a group of non-probability sampling techniques in which units are selected because they have characteristics that are needed in the sample. In this study, according to the typical types of grassland, combined with population, economic, and traffic conditions, the investigated towns (or “Sumu”, i.e., a town-level administration in North China) were determined. Within the four investigated banners that were considered to well represent all the land-use types in our study area, we selected towns and Sumu. In the investigated towns and Sumu, we also considered whether people primarily worked in the animal husbandry industry and the farming industry or the mixed animal husbandry industry and farming industry along with the corresponding conditions of the banner. 

In the second step, we determined the investigated villages or Gacha (i.e., a village-level administration in North China) by using the stratified random sampling method. This sampling method is able to ensure that every characteristic is properly represented in the sample, when a population’s characteristics are diverse. This will help with the generalizability and validity of the study, as well as avoiding research biases, such as under-coverage bias. Specifically, in this study, the investigated towns and villages were selected from the qualified towns/Sumu determined in the first step. In the end, we investigated ten Sumu in the typical pastoral grassland areas and four Sumu (towns) in the Hunshadake Sandyland pastoral area, as well as three towns in the agro-pastoral transitional zone as the study sites (Appendix A). 

In the final step, we adopted a random sampling method, in which each member of the population has an exactly equal chance of being selected. This is used to make statistical inferences about a population, and it helps to ensure high internal validity and external validity based on randomization. Considering the sparse population of the study sites (i.e., a population density of 0.2 person/km^2^) and the expansive distances between households, we considered the circumstances of the day on which we selected the farmers and herders for our field survey. During the survey, we randomly selected a route in the study area to conduct a questionnaire survey of farmers and herders who we met along the route, which made sure our interviewees’ determination followed the randomization principle. Approximately 60 farmer or herder households were given the questionnaire survey in each of the selected study sites. This provided us with a total of 238 farmer and herder households in 17 different towns (or Sumu) and 30 villages (or Gacha) to conduct our survey (Appendix A). There were 238 questionnaires and 230 valid questionnaires, with a 94.64% effective rate of the questionnaire. In total, the dataset involved information related to 652 family members among the 230 households gave support for further analyses. 

The collected dataset mainly included three information parts. The first part was the household demographic information, including family members’ genders, ages, education levels, ethnic groups, and relationships with the household head. The second part of the dataset involved the household characteristics, including family size, household rangeland, cropland size, and scale of livestock raised. The last part comprised household food consumption, including household food consumption types and food sources. 

During the survey, we first interviewed the household food consumption types over the past month. The data presented household food consumption status in 2019. Then, we asked the interviewees to recall their food consumption status during a typical day in 2009 and 1999. Along with the household food consumption status in 2009 and 1999, we obtained information regarding the household demographic and socioeconomic status at the same time. When the interviewees recalled their living conditions during different time periods, the information regarding household characteristics at the corresponding times (i.e., livestock raising scale, family income, family size, lifestyle, transportation conditions, market accessibility, and so on) helped them to draw a full picture of family food consumption status. Moreover, the results of this part were usually drawn from the discussions of all interviewed household’s family members. Based on their memories, families used the demographic and socioeconomic information to draw the typical daily food consumption status during the corresponding period. Although the accurate level of household food consumption could not be obtained, the interviewees admitted that the results corresponded to the remembered consumption levels in their memory. Thus, we believe the recalled results represented the conditions in 1999 and 2009. All these food consumption data formed the foundation of household dietary diversity-related analyses in this study. 

### 2.4. Data Analysis Methods

#### 2.4.1. Household Dietary Diversity Score

Household dietary diversity score (HDDS) was assessed by the household food consumption status using twelve food types, following the definitions of FAO [34]. As Table 1 shows, the twelve food types include: (1) cereals; (2) white tubers and roots; (3) legumes, nuts, and seeds; (4) vegetables; (5) fruits; (6) meat; (7) eggs; (8) fish and fish products; (9) milk and milk products; (10) alcohol; (11) oils and fats; and (12) spices, condiments, and beverages. The definitions of each type are also shown in the table. 

A score of 1 was assigned if household members had consumed a certain food belonging to a specific food type during the past month and 0 was assigned if no one had eaten said food item during the past month. The HDDS was calculated by adding up the scores that a household received for all twelve food types. 

Furthermore, we categorized the HDDS into two parts, namely animal-based food and plant-based food, by distinguishing the food sources. Specifically, plant-based food consisted of seven food types: cereals; white tubers and roots; legumes, nuts, and seeds; vegetables; fruits; alcohol; and spices, condiments, and beverages. The animal-based food included five food types: meat; eggs; fish and fish products; milk and milk products; oils and fats. Plant-based food scores and animal-based food scores were calculated by adding up the score distributions of the two kinds of food.

#### 2.4.2. Statistical Analytical Strategy

This study’s analyses were conducted in three parts. First, we performed a descriptive statistical analysis of rural residents’ food consumption evolution with a field survey collected dataset, which aimed at identifying the household consumption variation in twelve main food types among the four banners. Due to the limited data availability, we were not able to conduct the econometric analysis using household food consumption data but only using descriptive statistics. We also described the household demographic characteristics and household production activity-related characteristics. In particular, we developed two dummy variables to reflect the characteristics of the household head’s gender and ethnic group. For the variable “gender of the household head”, when the gender of the household head was female, a score of 1 was assigned, and 0 was assigned if the gender of the household head was male. The result revealed the percentage of households where a female was recognized as household head in our study sites. For the variable “ethnic group of the household head”, when the ethnic group of the household head was from Han ethnic group (which is the majority ethnic group in China), a score of 0 was assigned, and 1 was assigned if the ethnic group of household head was from a non-Han ethnic group. The result presented the percentage of households whose household head was from a minority (non-Han) ethnic group in our study sites. 

Second, we analyzed household dietary status over the past 20 years, including the data in 1999, 2009, and 2019. There are four analyses: household dietary diversity score, animal-based food consumption, and plant-based food consumption in 1999, 2009, and 2019, and average annual growth rate (AAGR) of households three types of scores. The equation to calculate the AAGR is as follows:

(1) $$R=\left(\sqrt[{n-1}]{S_{n}/S_{1}}-1\right)*100\%$$

where *R* represents average annual growth rate, *S* represents household dietary diversity score, and *n* represents the time period.

Third, a one-way analysis of variance (one-way ANOVA) was used to analyze the demographic characteristics, household characteristics, and HDDS features of sample households at all four study sites. The *p*-value < 0.05 was considered statistically significant. All analyses were performed using the software STATA^®^ version 15.1 (Stata Corporation, College Station, TX, USA) for Windows.

## 3. Results

### 3.1. Sample Characteristics

Table 2 shows the characteristics of the study samples. Among the 230 interviewed households, 31% of the household heads were female. The mean age of the household head was 51.14 (SD 12.21) years. More than half (55%) were from a minority ethnic group, while the remaining 45% were of Han ethnicity, which is the majority ethnic group in China. The household head received 7.46 (SD 3.74) years of school education on average. The family size in our study areas was relatively stable at around 3 people per family during 1999 to 2019, with a small scale of development from 2.93 to 3.01. Since household rangeland and cropland areas are quota-allocated and untradable in China, we reported both the size of household-owned land and the size of household-worked land. The results show that the household-owned rangeland size was 204.92 hectares, and the household-worked rangeland size was 195.52 hectares. Household-owned cropland size was 0.16 hectares, and the household-worked cropland size was 0.09 hectares. For the scale of livestock raised per household, both the cattle and sheep scales were decreased. Specifically, the cattle scale decreased from 40 to 26 from 1999 to 2019, and the sheep scale decreased from 304 to 179 from 1999 to 2019. 

Household demographic characteristics also show regional variations (Table 2). From north to south, the average age of household heads increased. Specifically, in the northern areas, such as East Ujimqin and West Ujimqin, the average age of household heads was 46.14 (SD 8.14) and 42.86 (SD 8.01) years, respectively. However, in the southernmost banner, Taibus, the average age of household heads was 62.98 (SD 9.30) years. For school education level, the household heads living in pastoral areas, such as East Ujimqin (7.30 years), West Ujimqin (8.51 years), and Zhenglan (8.78 years), had higher education levels than those living in agro-pastoral transition areas, such as Taibus (4.98 years). Regarding ethnic groups, the households living in the pastoral areas comprised mainly people of minority ethnic groups (mainly the Mongolian ethnic group in our study areas). In East Ujimqin, 91% of household heads were of non-Han ethnic groups. In West Ujimqin and Zhenglan, the proportion of non-Han minority ethnic groups was 86% and 61%, respectively. In comparison, all the household heads in Taibus were of the majority Han ethnic group. 

The household characteristics show regional variations as well (Table 2). The results of one-way ANOVA support the variations among the four banners, in which the differences in demographic and household characteristics among the four banners show significant differences (*p* < 0.001), except for the number of household cattle raised in 1999. In terms of family size, households in pastoral areas comprised bigger family sizes than those in agro-pastoral transition areas, and this feature has not changed during the past 20 years. Regarding household farmland, households in pastoral areas usually owned their own rangeland. Furthermore, they worked more rangeland than they owned by renting rangeland from other herder families or local cooperations. Specifically, the households in East Ujimqin, West Ujimqin, and Zhenglan worked 596.36 hectares, 184.01 hectares, and 84.79 hectares of rangeland, respectively. In contrast, the households in Taibus worked cropland only, with 0.59 hectares per family. In terms of the scale of livestock raised, there was a higher preference toward sheep than cattle. Households in pastoral areas tended to raise large numbers of sheep and cattle. However, sheep numbers decreased over the past 20 years except for in Taibus, because of the local environmental protection policies in the main grassland areas, such as in East Ujimqin, West Ujimqin, and Zhenglan. Due to the large scale of livestock raised, land degradation was severe in our study areas [35]. Thus, the local government encourages herder families to raise more cattle instead of sheep, especially in Zhenglan (the sandy land pastoral area). Moreover, compared to no families raising livestock in 1999, households in Taibus have since started to raise livestock, such as sheep and cattle, beginning in 2009 or even earlier.

### 3.2. Household Dietary Diversity Status

Table 3 shows the household dietary diversity status in 1999, 2009, and 2019. The results show that HDDS increased from 3.74 in 1999 to 5.92 in 2019, with an AAGR of 2.45% over the past 20 years in the herder community in the Xilin Gol Grasslands of China. Both animal-based food scores and plant-based food scores have increased, from 1.73 to 2.65 and 2.01 to 3.27, respectively. Plant-based food scores (AAGR 2.59%) have improved more than animal-based food scores (AAGR 2.29%). This result indicates that household dietary diversity has been improved, and plant-based food still remains the majority component of HDDS in the herder community in Xilin Gol. 

There are regional variations among the four banners. In general, HDDS is relatively higher in pastoral areas (East Ujimqin, West Ujimqin, and Zhenglan) than in agro-pastoral transition areas (Taibus), and this trend has been maintained from 1999 to 2019 (Table 3). We also used one-way ANOVA to test the difference between four banners, and the results support this variation. Both the *p*-value of HDDS and animal-based food score among four banners show significant differences (*p* < 0.05) (Table 3). 

However, regarding the improvement of household dietary diversity status, the household results in agro-pastoral transition areas are higher than those of households in pastoral areas. The results in Table 3 show that the AAGR of HDDS, animal-based food score, and plant-based food score all are highest in Taibus (except the plant-based food score, which was the second highest). The results indicate that, although household dietary diversity status in agro-pastoral transition areas has lagged behind households in pastoral areas, they have caught up with a high growth rate over the past 20 years.

Furthermore, the changes in the HDDS also indicate the residents’ lifestyle changes over the past 20 years. On the one hand, the increase in the HDDS means that people in the herder community are changing their daily food consumption to a more diverse one. On the other hand, the limited increase in animal-based food and the constant increase in plant-based food indicate that people tend to have a more balanced diet structure. 

## 4. Discussion

### 4.1. Household Food Consumption Impact Factors in Study Areas

Our results showed that HDDS increased over the past 20 years in Xilin Gol. The reasons that lead to these changes include a variety of impact factors. Among these factors, socio-economic development, ecological conditions, regional culture, and demographic factors are recognized as the main factors [21,36,37,38,39]. 

Specifically, the Xilin Gol Grassland is a typical grassland area, and local household food resources mainly depend on household production activities, such as raising sheep and cattle. Self-supply is the main source of household animal-based food supplies. While plant-based food is hard to obtain due to limited regional land resources. The results in Table 4 are consistent with our results in Table 2, indicating that although the scale animal raising per household decreased in grassland areas during the past 20 years, each family still has high levels of livestock. Household livestock-raising activities ensure household food security regarding meat supply. In our study, the animal-based food scores have had a consistent increase over the past 20 years. This strongly supports the above points.

Furthermore, the per capita annual income of rural residents has increased over five times in the past 20 years, while family size has become smaller (Table 4). Adequate budgets provide more possibilities for diversified household food consumption [40]. A more compact family size provides more possibilities to obtain and share more food within the family. The results of our study revealed that all the HDDS, animal-based food scores, and plant-based food scores at all study sites in Xilin Gol Grassland consistently increased in the past 20 years, which may be partially due to these factors.

Moreover, in herder communities, many traditional dishes are made from beef and mutton. With sufficient meat supply and budgets, households tend to consume more beef and mutton than ever before, which also promotes animal-based food consumption in the study areas. Our findings that animal-based food scores have had a consistent increase in the past 20 years are consistent with this.

### 4.2. Influences of Household Dietary Patterns

With socioeconomic development, residents’ food consumption structure has undergone great changes in Xilin Gol. Food intake is more diversified, and more animal-based food is being consumed than before. However, the intake of high-protein and high-fat food also brings health problems to residents. Especially in pastoral areas, since around 2000, the herder community has changed their lifestyle from a nomadic one to a settled one [41]. Therefore, herders do not need to move and migrate a long way in their daily life. Daily energy demand is thus less than it was before. However, the preference for beef and mutton in these areas has been retained [42]. Local rural residents were reported to be at much higher risk of hypertension and other non-communicable diseases [42,43]. Moreover, high animal-based food intake also reveals the environmental carrying capacity, and it could be a risk to the local environment since Xilin Gol Grassland is one of the most vulnerable areas in northern China [36]. Thus, in the future, policymakers should pay more attention to the health problems and environmental problems linked with household dietary patterns.

In this study, we did not obtain the seasonal variation of household dietary status, due to the limitations of our dataset, which was generated from the limited data collection activities. However, seasonal changes in household daily food should not be ignored [44,45]. The seasonal variations of household dietary status depend on more seasonal data collection, which is restricted by the budget and feasibility of local surveys. We will attempt to record the seasonal features of household dietary patterns in our study areas in the future.

### 4.3. Limitations of Our Study

We acknowledge four aspects of limitations in our studies. First, there is a recall bias in the dataset. We do not deny the existence of recall bias in this study. On the one hand, for household dietary status, we collected the household diet data for the past month during a field survey. Thus, the data have recall bias and may not 100% reflect the real household food consumption status [46,47]. On the other hand, for household food consumption status in 2009 and 1999, the data were collected through interviewee’s recall abilities, based on their memories and the household farming status of the corresponding time periods. They recalled the typical household dietary status, and thus we could not avoid recall bias. However, we attempted to use the recalled data to depict household dietary status, and multiple sampling methods helped us to make sure the samples (n 230) well represented the study areas. Although the results may not reflect an accurate number, we believe the results can provide us with some basis to understand the development and trends of household dietary status in the study areas. 

Second, our study only assessed the household food consumption status in different food groups via the dietary diversity score. Although we further assessed animal-based food scores and plant-based food scores to explore the different food sources, the changes in residents’ nutritional status and health status in the past 20 years could not be assessed. Previous studies revealed that more diverse dietary intake could contribute to people’s nutritional and health status. However, in this study, it is not possible to conclude changes related to nutritional and health status due to the limitation of the dataset.

Third, the cross-sectional data in this study cannot support the causal analyses to explore the reasons behind household food consumption patterns and their development. Additionally, during the data collection stage, the causal analyses demand was not met, which restricted our further analyses. We will attempt to collect more variables and information in future studies. 

Finally, due to the data accessibility and the particularities of the herder community, our results and findings could be hard to adapt to other cultivated farming communities. However, in this study, we explored household dietary diversity changes over the past 20 years. The results, based on first-hand data, spanned 20 years of changes and depicted the HDDS changes from when the community began changing their lifestyle from a nomadic one to a settled one. The results still have some scientific basis for regional development.

## 5. Conclusions

Household dietary diversity status has been improved in the herder communities of Xilin Gol Grassland in the past 20 years. The increase in HDDS is contributed to by both animal-based food and plant-based food, though the latter claimed the main role. The variation in HDDS between pastoral areas and agro-pastoral transition areas indicated that local food supply can highly affect household food consumption. 

Policymakers should pay attention to local lifestyle changes, including household dietary structures, local dietary demand, and dietary balance, while providing guidance and interventions for developing healthy dietary patterns. Furthermore, monitoring the main impact factors affecting HDDS will benefit local residents’ welfare and the local ecosystem. 

## Figures and Tables

**Table 1:** Component of household dietary diversity score (HDDS).

	Type	Definitions
1	Cereals	Bread, noodles, and other made from grains (including wheat, maize, rice, sorghum, or other local grains)
2	White tubers and roots	Potatoes, cassava, or other tubers
3	Legumes, nuts, and seeds	Cowpeas, peanuts, dried beans, dried peas, lentils, seeds, or foods made from these
4	Vegetables	Pumpkin, carrots, squash; any dark green leafy vegetables
5	Fruits	Mangoes, papayas, other vitamin A fruits; any other fruits
6	Meat	Meat (beef, pork, lamb, chicken, etc.), organ meat (liver, heart, other organs)
7	Eggs	Eggs from chicken, duck, or any other egg, other egg products
8	Fish and fish products	Sea fish, river fish, and other fish products
9	Milk and milk products	Cheese, yogurt, and other milk products
10	Alcohol	Beer, wine, and other alcohol
11	Oils and fats	Oil, fats, butter, and products made of these
12	Spices, condiments, and beverages	Tea, soft drinks, and other beverages

**Table 2:** Descriptive statistics of sample sizes and households.

	All Sample		East Ujimqin		West Ujimqin		Zhenglan		Taibus		*p*-Value
	(N = 230)	S.D.	(N = 49)	S.D.	(N = 57)	S.D.	(N = 62)	S.D.	(N = 62)	S.D.	
**Demographic characteristics**											
Gender of household head (1 = female)	0.31	0.46	0.08	0.28	0.32	0.47	0.31	0.46	0.44	0.50	0.003 **
Age of household head, years	51.14	12.21	46.14	8.14	42.86	8.01	49.87	10.99	62.98	9.30	<0.001 **
Education level of household head, years	7.46	3.74	7.30	3.23	8.51	3.30	8.78	3.04	4.98	4.03	<0.001 **
Ethnic group of household head (1 = non-Han)	0.55	0.50	0.91	0.28	0.86	0.35	0.61	0.49	0.00	0.00	<0.001 **
**Household characteristics**											
Family size in 2019, number of members	3.01	1.42	4.06	1.24	3.12	1.36	3.03	1.32	2.26	1.28	0.005 **
Family size in 2009, number of members	3.15	1.79	3.31	2.36	3.61	1.82	3.45	1.38	2.29	1.27	<0.001 **
Family size in 1999, number of members	2.93	1.63	2.88	1.96	3.37	1.69	3.23	1.37	2.26	1.29	<0.001 **
Household-owned rangeland size, ha	204.92	303.65	569.33	454.02	141.44	77.40	77.97	57.88	0.00	0.00	<0.001 **
Household-worked rangeland size, ha	195.52	387.16	596.39	657.45	184.01	200.53	84.79	71.71	0.00	0.00	<0.001 **
Household-owned cropland size, ha	0.16	0.38	0.00	0.00	0.00	0.00	0.01	0.08	0.59	0.52	<0.001 **
Household-worked cropland size, ha	0.09	0.29	0.00	0.00	0.00	0.00	0.01	0.08	0.31	0.50	<0.001 **
Cattle raised scale, number											
2019	25.85	31.22	38.38	47.60	26.58	29.02	32.60	22.26	2.06	2.86	<0.001 **
2009	22.72	24.77	24.21	31.67	23.10	24.75	30.98	19.67	2.50	6.62	<0.001 **
1999	40.10	50.87	51.25	45.89	57.77	69.80	20.50	14.95	0.00	0.00	0.147
Sheep raised scale, number											
2019	178.98	234.03	447.94	271.73	224.35	179.97	28.91	29.58	27.61	125.54	<0.001 **
2009	231.04	289.39	548.00	404.05	243.71	165.35	73.86	51.98	10.54	24.59	<0.001**
1999	303.60	304.68	643.33	319.17	385.00	235.32	86.25	85.58	0.00	0.00	<0.001**

Source: Authors’ survey conducted in 2019.Note: *p*-value shows the results of one-way ANOVA among four banners. ** represents statistical significance at the 1% level.

**Table 3:** Household Dietary Diversity Score (HDDS).

	All Samples	East Ujimqin	West Ujimqin	Zhenglan	Taibus	*p*-Value
**2019**						
HDDS	5.92	5.71	6.56	6.47	4.95	0.042 *
Animal-based food score	2.65	2.45	2.91	3.08	2.15	0.005 **
Plant-based food score	3.27	3.27	3.65	3.39	2.81	0.164
**2009**						
HDDS	4.58	4.47	5.39	4.87	3.65	0.009 **
Animal-based food score	2.09	1.92	2.42	2.44	1.56	<0.001 **
Plant-based food score	2.50	2.55	2.96	2.44	2.08	0.061
**1999**						
HDDS	3.74	3.73	4.12	4.19	2.94	0.015 *
Animal-based food score	1.73	1.61	1.98	2.08	1.23	<0.001 **
Plant-based food score	2.01	2.12	2.14	2.11	1.71	0.274
**1999–2019 AAGR**						
HDDS	2.45	2.26	2.48	2.31	2.79	N/A
Animal-based food score	2.29	2.22	2.04	2.09	2.99	N/A
Plant-based food score	2.59	2.29	2.85	2.51	2.64	N/A

HDDS: household dietary diversity score. AAGR: annual average growth rate. N/A: not applicable. Note: the *p*-value shows the results of one-way ANOVA in the four banners. ** and * represent statistical significance at the 1% and 5% levels.

**Table 4:** Regional statistical socioeconomic characteristics.

	Xilin Gol	East Ujimqin	West Ujimqin	Zhenglan	Taibus
**Family size, number of members**					
2019	2.28	2.39	2.34	2.18	2.05
2009	2.70	2.89	2.97	2.75	2.35
1999	3.23	3.36	3.67	3.30	3.17
Difference between 1999–2019	−0.95	−0.97	−1.33	−1.12	−1.12
1999–2019 growth rate (%)	−29.41	−28.87	−36.24	−33.94	−35.33
**Per capita livestock, number**					
2019	6.52	15.69	18.20	4.88	1.19
2009	7.06	23.93	12.78	4.42	0.61
1999	12.86	38.26	25.75	8.10	1.01
Difference between 1999–2019	−6.33	−22.57	−7.5	−3.23	0.18
1999–2019 growth rate (%)	−49.25	−58.99	−29.30	−39.82	17.76
**Per capita annual income of rural residents, CNY**					
2019	17,391	32,616	28,138	19,480	12,956
2009	5417	9997	8249	5954	4659
1999	2383	4956	3855	2008	1396
Difference between 1999–2019	15,008	27,660	24,283	17,472	11,560
1999–2019 growth rate (%)	629.79	558.11	629.91	870.12	828.08

Source: Xilin Gol League Statistics Yearbook (1999–2019). Note: The per capita livestock number in the statistical yearbook show the number of livestock at the end of the year. Our field survey showed the livestock number in the middle of the year, and herders usually sell livestock in the middle of the year. Thus, the statistical values of the two were not consistent.

## Data Availability

The data presented in this study are available upon request from the corresponding author.

## Appendix A

**Table A1:** Information on investigated villages.

Banner	Town or Sumu	Village or Gacha	Number of Interviewees
East Ujimqin	Gadabuqi Town Manduhubaolage Town Huritu Sumu Ejinaoer Sumu Uragai Area	Bayandulan Gacha Mandu Gacha, Manduhubaolage Gacha Chagannaoer Gacha Mulehumudele Gacha Uragai pastoral zone, Nongnai pastoral zone, Bulin pastroal zone	49
West Ujimqin	Jirengaole Town Wulanhalaga Sumu Bayanhushu Sumu Bayanhua Town Haoletugaole Town	Shandanbaolige Gacha Xingaole Gacha Saihannuoer Gacha Bayanhaoletu Gacha Wuritugaole Gacha, Alatangaole Gacha, Jinhe Gacha	57
Zhenglan	Sanggendalai Town Shangdu Town Saiyinhuduga Sumu Naritu Sumu	Bayinsurige Gacha Qinggeletu Gacha, Yirilejihu Gacha Chaidamu Gacha, Tugurige Gacha Ligesitai Gacha, Hulusitai Gacha	62
Taibus	Yongfeng Town Qianjingou Town Baochang Town	Tianxingyuan Village, Hujiaying Village Pingchuan Village Fuxing Village, Liuyuanying Village, Wodi Village, Fanmao Village, Zhengjiaying Village	62
Total	17	30	230
